# Residential Proximity to Major Roadways and Lung Cancer Mortality. Italy, 1990–2010: An Observational Study

**DOI:** 10.3390/ijerph13020191

**Published:** 2016-02-03

**Authors:** Ettore Bidoli, Marilena Pappagallo, Silvia Birri, Luisa Frova, Loris Zanier, Diego Serraino

**Affiliations:** 1 Unit of Epidemiology and Biostatistics, Centro di Riferimento Oncologico, IRCCS, Aviano 33081, Italy; birris@cro.it (S.B.); serrainod@cro.it (D.S.); 2 Division for Socio-Demographic and Environmental Statistics—Statistics on Health and Social Security, National Institute of Statistics, Rome 00184, Italy; pappagal@istat.it (M.P.); frova@istat.it (L.F.); 3 Direzione Centrale Salute, Friuli Venezia Giulia, Servizio Regionale di Epidemiologia, Udine 33100, Italy; loris.zanier@regione.fvg.it

**Keywords:** lung cancer, mortality, residence proximity, major roadways, Italy, nationwide

## Abstract

Background: Air pollution from road traffic has been associated to an increased risk of lung cancer. Herein, we investigated the association between lung cancer mortality and residence near Italian highways or national major roads. Methods: Information on deaths for lung cancer registered from 1990 to 2010 and stratified by age, gender, and urban or rural municipality of residence at death were obtained from the National Institute of Statistics. Distance between the centroid of the municipality of residence and closest major roadways was considered as a proxy of pollution exposure. Relative Risks (RR) and 95% confidence intervals (CI) were computed using Poisson log-linear models adjusted for age, calendar period, deprivation index, North/South gradient, and urban/rural status. Results: A gradient in risk for lung cancer mortality was seen for residents within 50 meters (m) of national major roads. In particular, in rural municipalities a statistically significant increased risk for lung cancer death was observed in both sexes (RR = 1.27 for distance <25 m *vs.* 500–1999 m, 95% CI 1.17–1.42, in men; RR = 1.97, 95% CI 1.64–2.39, in women). In urban municipalities, weak risks of borderline significance were documented in both sexes (RR = 1.06, 95% CI 0.99–1.15 in men; and RR = 1.09, 95% CI 0.97–1.22 in women). No statistically significant association emerged between residence within 100 to 500 m from highways and RRs of death for lung cancer. Conclusions: In Italy, residing near national major roads, in particular in rural municipalities, was related to elevated risks of death for lung cancer.

## 1. Introduction

Besides cigarette smoking, outdoor air pollution (OAP) has been associated with an increased risk of lung cancer. The International Agency for Research on Cancer (IARC) reviewed, in 2013, the epidemiological and mechanistic evidence regarding the association between OAP and the risk of lung cancer. The main conclusion of the review was a sufficient evidence that exposure to OAP, particularly to particulate matter (PM) is a Group 1 carcinogen (IARC classification) to humans and that it causes lung cancer [1,2].

Heavy traffic roads have been considered an independent source of OAP linked to elevated lung cancer risk [3,4,5,6,7,8,9,10]. Studies conducted mainly in large cities and their surroundings found a direct association between lung cancer risk and residential proximity to major roadways, though the magnitude of the risk estimates varied among studies. One potential explanation of the different magnitude of the risks may be the heterogeneity in the type of exposure considered across studies (for instance, a different urbanization of the cities and/or in the types of roads included). Nevertheless, the studies that quantified the concentration of pollutants near roadways showed that the concentration of both gaseous pollutants and PM were higher in a range of 200 to 500 meters (m) closer to major roads [4,8,9,10,11,12,13,14].

Further studies that stratify lung cancer risk by urban/rural (UR) residence and by type of major roadways, *i.e.*, highways and national major roads, are needed to give additional clues about the magnitude of the relationship between lung cancer risk and traffic-related pollution.

To shed further light on this topic, we conducted a nationwide observational study to quantify the association between risk of death for lung cancer and traffic-related air pollution. The study was carried out in 1515 Italian urban and rural municipalities whose centroid was within 1999 m from major roadways.

## 2. Methods

Information regarding the underlying cause of death in individual death certificates was extracted from the electronic database of the Italian National Institute of Statistics (ISTAT). Two different revisions of the International Classification of Diseases (ICD) were used to code the underlying cause of death during the study period, *i.e.*, 1990–2010, the ninth revision from 1990 to 2002; and the 10th revision since 2003. For the aims of this analysis, we considered 153,892 deaths attributed to lung cancer (126,350 in men, 27,542 in women), recoded according to the ICD-10 (C33–C34). 

The 1990–2010 Italian resident population (approximately 57 million inhabitants, yearly) was abstracted from the ISTAT database. Deceased and resident population were stratified by age (in quinquennia), sex, calendar period, and municipality of residence. The coding of causes of death were not completed at the time of this analysis for the years 2004 and 2005, thus such years were excluded from the two calendar periods (*i.e.*, 1990–2003 and 2006–2010) used in the analysis.

We separately considered Italian highways and national major roads (including collector roads (according to the definitions of the Italian national road works company—ANAS)). In particular, highways were main roads for heavy and fast-moving traffic, with limited access, separate carriageways for vehicles travelling in opposite directions, at least two lanes each direction, and they are generally located away from populated areas. National major roads are roadways connecting main municipalities, having at least one lane for each direction, stop lights, intersections, and roundabouts (http://www.stradeanas.it/). All highways and national major roads had been already in use since the 1970s.

An ecological approach for the whole of Italy was adopted by using group-based metrics and crude confounders, with the municipality of residence as unit of analysis. Moreover, it should be noted that, in the death certificate, the residential street address within each municipality and the duration of residence were not available. Therefore, we used the centroid of the municipality of residence at death abstracted from ISTAT death certificates as a proxy indicator of long term exposure to traffic-related air pollution. 

The level of exposure was approximated by measuring the shortest distance between the centroid of each municipality and the major roadways by means of the Geographic Information System (GIS) (GeoMedia Professional V 6.1, 1996–2007). For national major roads, the distance in meters was recoded into six intervals according to the international literature (<25 m; 25–49 m; 50–74 m; 75–99 m; 100–499 m; and 500–1999 m). Due to the low number of municipalities within 100 m of highways (*N* = 7, near 11,000 inhabitants), we a priori recoded distance into three intervals (<100 m; 100–499 m; and 500–1999 m). The list of all Italian major roads can be inferred from (http://www.stradeanas.it/). As a result of this selection, 1515 municipalities out of 8048 Italian municipalities (18%) were involved in the study, with 18.7 million inhabitants (*i.e.*, 33% of the Italian population). Municipalities with centroid within 1999 m from national major roads were 939 (yearly population of 9.8 million inhabitants) while those with centroid within 1999 m from highways were 576 (yearly population of 8.9 million inhabitants). Moreover, 40% of the 1515 municipalities were urban municipalities.

Nationwide data at the individual level concerning well-known risk factors for lung cancer (e.g., smoking) are not available. Thus, as potential confounders defined on a priori evidence, we considered the deprivation index (DI) [15], the geographic North-South gradient according to a standard definition (www.registri-tumori.it), and the UR status [16]. In brief, DI is based on five indicators (low education, unemployment, residential tenancy, absence of indoor sanitary facilities, and single parenthood), and it is classified in quintiles with the first level representing the best socio-economic status and the last the worst one. The DI has been strongly associated to smoking with divergent patterns in men and women in Italy during the last two decades of the XXth century [17,18]. In fact, the higher prevalence of smokers was associated with low educational levels in men and with high educational levels in women [18]. Concerning the North-South gradient, since the 1970s in both sexes, lung cancer mortality rates were more elevated in the northern regions of Italy as compared to southern ones, with intermediate rates in central areas-. For instance, in the mid-1970s, the North/South mortality rate ratio was around 1.5 (www.registri-tumori.it) [19].

### Statistical Analysis

We quantified the effect of municipality of residence near highways and national major roads on the risk of death for lung cancer by means of relative risks (RRs) and corresponding 95% confidence intervals (CI), using Poisson log-linear models (SAS 9.20, SAS Institute Inc., Cary, NC, USA). All RRs were adjusted for age, calendar period, DI, north-south gradient, and when appropriate, UR status. The models were also stratified by type of major roadways, sex, UR status, and calendar period. The dependent variable was the number of lung cancer deaths, offset by population. Log-linear models were fitted on the assumption that the number of deaths per stratum followed a Poisson distribution. In tables, we also listed RRs for UR status, DI and North-South gradient, derived from the multivariate models in order to fit the a priori evidence about lung cancer distribution in Italy.

## 3. Results 

Table 1 lists RRs for lung cancer mortality according to sex and UR status by selected variables and municipality proximity to national major roads. Overall, statistically significant elevated risks of death for lung cancer in municipalities within 25 m of national major roads were recorded in both men (RR = 1.10 for distance <25 m *vs.* 500–1999 m, 95% CI 1.06–1.14) and women (RR = 1.25, 95% CI 1.10–1.44). Likewise, residing in municipalities between 25–49 m was also associated with an elevated risk in men (RR = 1.05 for distance between 25–49 m *vs.* 500–1999 m, 95% CI 1.01–1.06), and in women (RR = 1.12, 95% CI 1.06–1.23). Results from the stratified analysis according to UR residence highlighted statistically significant elevated risks of death for lung cancer for residency in rural municipalities. As compared to those residing in rural municipalities located 500 m to 1999 m from national major roads, RRs for lung cancer death in women residing within 50m were 1.97-fold (<25 m) or 1.16-fold (25–49 m) higher. In men, these RRs were significantly elevated only for those residence <25 m (1.27, 95% CI: 1.17–1.42) (Table 1). Conversely, residence in urban municipalities located <25 m from national major roads turned out to be associated with a weak elevated RR of borderline significance in both sexes (*i.e.*, 1.06, 95% CI 0.99–1.15 in men; and 1.09, 95% CI 0.97–1.22 in women). 

Table 1 shows RRs for lung cancer mortality related to other selected variables. An overall excess risk was documented in rural municipalities -as compared to urban ones (RR = 1.17 for rural *vs.* urban, 95% CI 1.14–1.20, in men; and RR = 1.50, 95% CI 1.42–1.58, in women). The worst DI (*i.e.*, the fifth quintile) was related to an elevated risk of lung cancer mortality in males (all municipalities combined, RR = 1.22 for high DI *vs.* low DI, 95% CI 1.19–1.25). Conversely, an inverse association was observed among women (RR = 0.87, 95% CI 0.82–0.92) (Table 1). Furthermore, an excess of mortality was recorded in northern Italy, as compared to the southern part (all municipalities combined, RR = 1.22 for North *vs.* South, 95% CI 1.19–1.25, in men; and RR = 1.57, 95% CI 1.48–1.66 in women) (Table 1). 

The risk of death for lung cancer according to sex, and UR status by selected variables and municipality proximity to highways is shown in Table 2. No gradient in the risk of death for lung cancer was observed for people residing in municipalities located <500 m from highways. Conversely, RRs for deaths due to lung cancer associated to UR status, DI, and North-South gradient were substantially similar to those observed for national major roads and described in Table 1.

The relation between lung cancer mortality and residential proximity to major roadways was further examined in strata of age (<65 and ≥65 years), North-South gradient, and calendar period (1990–1999 and 2000–2010) (data not shown). Although some differences in RRs were observed across strata of age, north-south gradient, or the two calendar periods, they were compatible with random variation since the tests for interaction across strata were not statistically significant. For instance, in rural municipalities within 25 m of national major roads, a statistically significant risk was observed in both the <65 years age group (RR = 1.57, 95% CI 1.38–1.82 in men, and RR = 2.79, 95% CI 1.97–3.76 in women) and the ≥65 years age group (RR = 1.15, 95% CI 1.04–1.23 in men, and RR = 1.77, 95% CI 1.44–2.18 in women).

## 4. Discussion 

Our investigation, the first using a comprehensive Italian multi-city approach, showed a 16% to 97% increased risk for lung cancer deaths in rural municipalities whose centroid was within 50 m from national major roads, as compared to those with a centroid included in the range 500–1999 m. No similar magnitude in risk was observed in urban municipalities (with RRs of borderline statistical significance), neither in municipalities located <500 m from highways. Given a 20–40 years latency period between exposure and diagnosis of lung cancer, these results indicate that, in Italy, exposure to pollution from road traffic in rural municipalities located close to national major roads from the 1970s to the 1980s substantially increased the risk of death for lung cancer. 

Moreover, these results may suggest that the health effects in Italian rural municipalities near national major roads may be larger than previously quantified in metropolitan areas. A plausible explanation is that urban pollution, or unmeasured confounding linked to urbanization, may distort a precise quantification of risk linked to traffic-related air pollution. In particular, it has been shown that smoking (the well-known major risk factor for lung cancer) was more prevalent in urban than in rural areas [4]. Accordingly, it is likely that the ecological adjustment carried out in this study may have left some residual confounding of smoking in urban areas. 

In addition, our study broadly confirmed the a priori evidence, in Italy, regarding the association between lung cancer, DI, and the North/South gradient. Concerning DI, it is worth noting that it is inversely related with educational level and directly associated with smoking. To this regard, in the last two decades of the XXth century, the prevalence of smoking in Italy was higher in men (56%) than in women (18%), more frequently so in less educated men and in more educated women [19].

A major finding of this investigation was that the association between road proximity and risk of death for lung cancer was more prominent in women than in men. In our opinion, this observation can be explained, at least partially, by the overall lower impact of smoking on lung cancer risk in women than in men. In addition to the different patterns of smoking in men and women according to SES and DI, the overall prevalence of smoking was, in the 1970s–1980s, lower in Italian women (18%) than in Italian men (56%) [19]. In this scenario, it is likely that the role of air pollution on lung carcinogenesis recently documented by the IARC [2] may have a higher impact in women than in men.

**Table 1:** Relative risks **^a^** of death for lung cancer in 939 municipalities by proximity to national major roads (Italy, 1990–2010).

Variables	Men						Women					
	Rural + Urban		Rural		Urban		Rural + Urban		Rural		Urban	
	RR	(95% CI)	RR	(95% CI)	RR	(95% CI)	RR	(95% CI)	RR	(95% CI)	RR	(95% CI)
Urban/rural status												
Urban	1		-		-		1		-		-	
Rural	1.17	(1.14–1.20)	-	-	-	-	1.50	(1.42–1.58)	-	-	-	-
Deprivation Index (quintiles)												
1—Best	1		1		1		1		1		1	
2	1.07	(1.05–1.10)	1.07	(1.01–1.14)	1.07	(1.04–1.10)	0.87	(0.83–0.91)	0.74	(0.65–0.84)	0.90	(0.85–0.95)
3	1.11	(1.07–1.15)	1.02	(0.95–1.09)	1.21	(1.16–1.26)	0.99	(0.92–1.08)	0.68	(0.57–0.80)	1.18	(1.08–1.30)
4	1.06	(1.03–1.09)	1.07	(1.00–1.14)	1.08	(1.04–1.11)	0.87	(0.82–0.93)	0.64	(0.55–0.76)	0.94	(0.88–1.01)
5—Worst	1.22	(1.19–1.25)	1.32	(1.23–1.60)	1.17	(1.14–1.21)	0.87	(0.82–0.92)	0.79	(0.67–0.92)	0.86	(0.80–0.92)
North/South gradient												
South	1		1		1		1		1		1	
Center	1.07	(1.04–1.10)	1.22	(1.14–1.29)	1.00	(0.97–1.04)	1.09	(1.02–1.17)	1.21	(1.05–1.40)	1.01	(0.94–1.09)
North	1.22	(1.19–1.25)	1.46	(1.38–1.54)	1.14	(1.11–1.17)	1.57	(1.48–1.66)	1.78	(1.55–2.03)	1.45	(1.37–1.55)
Residence proximity to national major roads (m)												
500–1999	1		1		1		1		1		1	
100–499	0.98	(0.96–1.01)	1.02	(0.97–1.05)	0.96	(0.94–1.02)	1.04	0.98–1.12)	1.07	(0.95–1.18)	1.00	(0.95–1.06)
75–99	0.99	(0.94–1.05)	1.01	(0.93–1.04)	0.97	(0.95–1.03)	1.05	(0.94–1.15)	0.99	(0.75–1.26)	1.09	(0.96–1.25)
50–74	1.00	(0.97–1.07)	0.99	(0.91–1.11)	1.00	(0.94–1.06)	1.04	(0.94–1.14)	1.06	(0.88–1.29)	1.02	(0.93–1.14)
25–49	1.05	(1.01–1.06)	1.08	(0.99–1.29)	1.03	(0.98–1.07)	1.12	(1.06–1.23)	1.16	(1.01–1.33)	1.07	(0.96–1.19)
<25	1.10	(1.06–1.14)	1.27	(1.17–1.42)	1.06	(0.99–1.15)	1.25	(1.10–1.44)	1.97	(1.64–2.39)	1.09	(0.97–1.22)

**^a^** Estimates from multivariate models adjusted for age, calendar period, deprivation index, North/South gradient, and urban/rural status, when appropriate. RR = Relative Risk; CI = Confidence Interval.

**Table 2:** Relative risks **^a^** of death for lung cancer in 576 municipalities by proximity to highways (Italy, 1990–2010).

Variables	Men						Women					
	Rural + Urban		Rural		Urban		Rural + Urban		Rural		Urban	
	RR	(95% CI)	RR	(95% CI)	RR	(95% CI)	RR	(95% CI)	RR	(95% CI)	RR	(95% CI)
Urban/rural status												
Urban	1		-		-		1		-		-	
Rural	1.24	(1.21–1.27)	-	-	-	-	1.69	(1.59–1.80)	-	-	-	-
Deprivation Index (quintiles)												
1—Best	1		1		1		1		1		1	
2	1.05	(1.02–1.07)	1.13	(1.05–1.20)	1.06	(1.03–1.08)	0.90	(0.85–0.94)	1.11	(0.96–1.30)	1.90	(0.86–0.95)
3	1.07	(1.04–1.10)	1.24	(1.14–1.34)	1.07	(1.04–1.11)	0.86	(0.81–0.92)	1.38	(1.14–1.68)	0.85	(0.80–0.91)
4	1.04	(1.01–1.07)	1.32	(1.20–1.42)	1.00	(0.97–1.03)	0.88	(0.83–0.93)	1.51	(1.26–1.81)	0.86	(0.79–0.89)
5—Worst	1.17	(1.14–1.20)	1.30	(1.22–1.39)	1.16	(1.13–1.20)	0.90	(0.85–0.95)	1.21	(1.03–1.42)	0.88	(0.83–0.94)
North/South gradient												
South	1		1		1		1		1		1	
Center	1.08	(1.05–1.12)	1.38	(1.28–1.49)	0.98	(0.95–1.02)	1.23	(1.15–1.32)	1.86	(1.55–2.22)	1.06	(0.98–1.15)
North	1.34	(1.31–1.37)	1.86	(1.77–1.96)	1.23	(1.20–1.26)	1.73	(1.63–1.82)	2.10	(1.75–2.51)	1.49	(1.41–1.59)
Residence proximity to highways (m)												
500–1999	1		1		1		1		1		1	
100–499	1.05	(0.97–1.12)	1.07	(0.95–1.18)	1.01	(0.98–1.04)	0.99	(0.95–1.06)	1.04	(0.93–1.16)	0.98	(0.95–1.02)
<100	1.04	(0.92–1.23)	1.03	(0.90–1.22)	1.05	(0.89–1.27)	1.02	(0.92–1.24)	1.01	(0.88–1.26)	1.02	(0.95–1.19)

**^a^** Estimates from multivariate models adjusted for age, calendar period, deprivation index, North/South gradient, and urban/rural status, when appropriate. RR = Relative Risk; CI = Confidence Interval.

The findings from this study add to the evidence from previous investigations on the health effects of long-term exposure to air pollution on lung cancer risk [2]. Emphasis on these studies has been on different pollutants and on different exposure assessment methods such as aggregation of air pollution monitoring levels within cities or defined areas, estimation of environmental air pollution levels at residential addresses using fixed-site monitoring data or dispersion models, or use of road proximity as exposure surrogates. In particular, two studies have shown a statistically significant association between lung cancer risk and residence proximity to major roadways, one specifically in non-smokers [20], and one in women only [21]. In other studies, the calculation of the shortest distance between residence and major roadways was irregularly associated with lung cancer risk [4,8,9,10]. Finally, studies that examined various gaseous and particulate indicators of traffic pollution found a direct association with lung cancer risk [4,8,9,10,22,23]. In a prospective study conducted in never- or ex-smokers, the fraction of lung cancers attributable to high levels of pollution were conservatively estimated to be around 5%–7% [10]. A meta-analysis of cohort and case-control studies calculated a 15%–21% increase of lung cancer incidence associated with 10 ug/m^3^ increase of PM_2.5_ [7]. 

Mechanisms of distribution of gaseous and particulate pollutants around major roadways may explain the risk observed [11,12,13]. Sharp pollutant gradients exist near highways. In particular, particle number concentrations decreased nearly five-fold within 30 m of a major roadway [11] as for gaseous compounds. For instance, relative concentrations of carbon monoxide, black carbon, and total particle number concentration decreased exponentially between 17 and 150 m downwind from the highways, while at 300 m ultrafine particle concentrations were the same as at upwind sites [12]. Thus, people living close to highways are likely to receive a much higher exposure to traffic-related air pollutants compared to residents living at >200–250 m distance [11,13].

Our results have some worth noting limitations linked to the lack of individual-level data nationwide. Pollution exposure was approximated by calculating the shortest distance between the centroid of the municipality of the residential address and the closest high traffic road. Individual information on smoking habits was not available and as previously done, we adjusted the models for deprivation index which could be a predictor of smoking habits independent of personal characteristics [15,17]. Lung cancer takes many years to develop and residence at death is likely to be a poor measure of lifelong exposure to pollution. Nevertheless, ignoring latency should flatten the risk estimates if population commuting is random with respect to lung cancer. Moreover, residents in urban settings may move more frequently compared to residents in rural settings, which could attenuate effect estimates obtained in urban settings. Finally, emissions from vehicles may have changed significantly over time due to improved vehicle emission controls. 

Study strengths include the very large series of mortality data that allowed for stable risk estimates. Selection bias is unlikely, because we took advantage of the complete Italian official mortality database and we used underlying lung cancer death as the endpoint of the study. The effects of single air pollutants are difficult to disentangle, while distance is a comprehensive proxy of any pollutant exposure not susceptible to the potential bias of selecting indicators difficult to measure or not specifically linked to traffic exposure. We adjusted all models for UR status and North-South gradient to take into account urban and regional pollution nationwide. 

## 5. Conclusions

In conclusion, our results confirm previous evidence suggesting that dwelling in municipalities located within 50 m from national major roads is associated with an increased risk of death for lung cancer. They also show that—at least in ecological studies—the precision of the assessment of lung cancer risk in such setting may be improved by UR stratification.

## References

[1] Loomis D., Grosse Y., Lauby-Secretan B., El Ghissassi F., Bouvard V., Benbrahim-Tallaa L., Guha N., Baan R., Mattock H., Straif K. (2013). The carcinogenicity of outdoor air pollution. Lancet Oncol..

[2] Straif K., Cohen A., Samet J. (2013). Air Pollution and Cancer.

[3] Yorifuji T., Kashima S. (2013). Air pollution: Another cause of lung cancer. Lancet Oncol..

[4] Raaschou-Nielsen O., Andersen Z.J., Beelen R., Samoli E., Stafoggia M., Weinmayr G., Hoffmann B., Fischer P., Nieuwenhuijsen M.J., Brunekreef B. (2013). Air pollution and lung cancer incidence in 17 European cohorts: Prospective analyses from the European Study of Cohorts for Air Pollution Effects (ESCAPE). Lancet Oncol..

[5] Hamra G.B., Guha N., Cohen A., Laden F., Raaschou-Nielsen O., Samet J.M., Vineis P., Forastiere F., Saldiva P., Yorifuji T. (2014). Outdoor particulate matter exposure and lung cancer: A systematic review and meta-analysis. Environ. Health Perspect..

[6] Hoek G., Brunekreef B., Goldbohm S., Fischer P., Van den Brandt P.A. (2002). Association between mortality and indicators of traffic-related air pollution in the Netherlands: A cohort study. Lancet.

[7] Chen H., Goldberg M.S., Villeneuve P.J. (2008). A systematic review of the relation between long-term exposure to ambient air pollution and chronic diseases. Rev. Environ. Health.

[8] Cesaroni G., Badaloni C., Gariazzo C., Stafoggia M., Sozzi R., Davoli M., Forastiere F. (2013). Long-term exposure to urban air pollution and mortality in a cohort of more than a million adults in Rome. Environ. Health Perspect..

[9] Hystad P., Demers P.A., Johnson K.C., Carpiano R.M., Brauer M. (2013). Long-term residential exposure to air pollution and lung cancer risk. Epidemiology.

[10] Vineis P., Hoek G., Krzyzanowski M., Vigna-Taglianti F., Veglia F., Airoldi L., Overvad K., Raaschou-Nielsen O., Clavel-Chapelon F., Linseisen J. (2007). Lung cancers attributable to environmental tobacco smoke and air pollution in non-smokers in different European countries: A prospective study. Environ. Health.

[11] Brugge D., Durant J.L., Rioux C. (2007). Near-highway pollutants in motor vehicle exhaust: A review of epidemiologic evidence of cardiac and pulmonary health risks. Environ. Health.

[12] Zhu Y., Hinds W.C., Kim S., Sioutas C. (2002). Concentration and size distribution of ultrafine particles near a major highway. J. Air Waste Manag. Assoc..

[13] Padró-Martínez L.T., Patton A.P., Trull J.B., Zamore W., Brugge D., Durant J.L. (2012). Mobile monitoring of particle number concentration and other traffic-related air pollutants in a near-highway neighborhood over the course of a year. Atmos. Environ..

[14] HEI Panel on the Health Effects of Traffic-Related Air Pollution (2010). Traffic-Related Air Pollution: A Critical Review of the Literature on Emissions, Exposure, and Health Effects.

[15] Cadum E., Costa G., Biggeri A., Martuzzi M. (1999). Deprivation and mortality: A deprivation index suitable for geographical analysis of inequalities. Epidemiol. Prev..

[16] Bidoli E., Franceschi S., Dal Maso L., Guarneri S., Barbone F. (1993). Cancer mortality by urbanization and altitude in a limited area in northeastern Italy. Rev. Epidemiol. Sante Publ..

[17] Diez Roux A.V. (2003). Residential environments and cardiovascular risk. J. Urban Health.

[18] Facchini U., Camnasio M., Cantaboni A., Decarli A., La Vecchia C. (1985). Geographical variation of cancer mortality in Italy. Int. J. Epidemiol..

[19] Gorini G., Carreras G., Allara E., Faggiano F. (2013). Decennial trends of social differences in smoking habits in Italy: A 30-year update. Cancer Cause. Control.

[20] Raaschou-Nielsen O., Andersen Z.J., Hvidberg M., Jensen S.S., Ketzel M., Sørensen M., Loft S., Overvad K., Tjønneland A. (2011). Lung cancer incidence and long-term exposure to air pollution from traffic. Environ. Health Perspect..

[21] Visser O., Van Wijnen J.H., Van Leeuwen F.E. (2004). Residential traffic density and cancer incidence in Amsterdam, 1989–1997. Cancer Cause. Control.

[22] Beelen R., Hoek G., Van den Brandt P.A., Goldbohm R.A., Fischer P., Schouten L.J., Jerrett M., Hughes E., Armstrong B., Brunekreef B. (2008). Long-term effects of traffic-related air pollution on mortality in a Dutch cohort (NLCS-AIR study). Environ. Health Perspect..

[23] Yorifuji T., Kashima S., Tsuda T., Ishikawa-Takata K., Ohta T., Tsuruta K., Doi H. (2013). Long-term exposure to traffic-related air pollution and the risk of death from hemorrhagic stroke and lung cancer in Shizuoka, Japan. Sci. Total Environ..

